# Predicting Pathological Complete Response in Neoadjuvant Dual Blockade With Trastuzumab and Pertuzumab in HER2 Gene Amplified Breast Cancer

**DOI:** 10.3389/fimmu.2022.877825

**Published:** 2022-05-19

**Authors:** Yi Xiao, Jiahan Ding, Dachang Ma, Sheng Chen, Xun Li, Keda Yu

**Affiliations:** ^1^Department of Breast Surgery, The First Hospital of Lanzhou University, Lanzhou, China; ^2^Department of General Surgery, The First Hospital of Lanzhou University, Lanzhou, China; ^3^Department of Breast Surgery, Fudan University Shanghai Cancer Center, Shanghai, China

**Keywords:** trastuzumab, pertuzumab, predictive model, neoadjuvant treatment, HER2-amplified breast cancer

## Abstract

**Background:**

Dual-targeted therapy is the standard treatment for human epidermal growth factor receptor 2 (HER2)-positive breast cancer, and effective biomarkers to predict the response to neoadjuvant trastuzumab and pertuzumab treatment need further investigation. Here, we developed a predictive model to evaluate the dual-targeted neoadjuvant treatment efficacy in HER2 gene-amplified breast cancer.

**Method:**

This retrospective study included 159 HER2-amplified patients with locally advanced breast cancer who received neoadjuvant trastuzumab, pertuzumab, and chemotherapy. The correlation between clinicopathological factors and pathological complete response (pCR, in the breast and axilla) was evaluated. Patients were randomly assigned into the training set (n=110) and the testing set (n=49). We used an independent cohort (n=65) for external validation. We constructed our predictive nomogram model with the results of risk variables associated with pCR identified in the multivariate logistic analysis. The area under the curve (AUC) of the receiver operating characteristic (ROC) curve, decision curve analysis, and calibration curves were employed to assess the nomogram’s performance.

**Results:**

We revealed that the HER2/CEP17 ratio (*p=*0.001), CD8 levels (*p*=0.005), and histological grade (*p*=0.007) were independent indicators for pCR in dual-targeted neoadjuvant treatment after multivariate adjustment. The combined prediction efficacy of the three indicators was significantly higher than that of each single indicator alone. The AUCs were 0.819, 0.773, and 0.744 in the training, testing, and external validation sets, respectively.

**Conclusions:**

The HER2/CEP17 ratio, CD8 levels, and histological grade were significantly correlated with pCR in dual-targeted neoadjuvant treatment. The combined model using these three markers provided a better predictive value for pCR than the HER2/CEP17 ratio, CD8 levels, and the histological grade alone, which showed that an immunological effect partially mediates the predictive impact of neoadjuvant treatment.

## Introduction

The human epidermal growth factor receptor 2 (HER2) gene, which plays a pivotal part in tumor formation and growth processes, is amplified to roughly 20%–30% of all breast cancers (1). HER2 amplification can be defined by protein expression, HER2 gene copy number alterations, or the HER2/CEP17 (centromere enumerator probe 17) ratio (2). Immunohistochemistry (IHC) and fluorescence *in situ* hybridization (FISH) are widely used to define HER2 status according to the American Society of Clinical Oncology/College of American Pathologists (ASCO/CAP) guidelines for HER2 testing (3). HER2 gene amplification is associated with poor clinical outcomes (4, 5). Pathological complete response (pCR) of the tumor has been extensively used as the major endpoint in neoadjuvant chemotherapy (NAC) trials. It is an essential independent indicator in the prognosis of prolonged disease-free and overall survival time (6). Anti-HER2 therapy is currently the standard treatment for HER2-amplified patients in adjuvant and neoadjuvant settings. More recently, neoadjuvant treatment for HER2 gene-amplified locally advanced breast cancer patients has significantly expanded with the advent of dual-targeted treatment by trastuzumab and pertuzumab. The combination of trastuzumab with pertuzumab and standard chemotherapy confirmed the improvement of the pCR rate in the TRYPHAENA and NeoSphere studies (7, 8). The patients included in this study were consecutive incident cases who had HER2-amplified locally advanced breast cancer and had received six cycles of docetaxel and carboplatin (TCb) NAC with trastuzumab and pertuzumab.

Previous research confirmed that a higher pCR rate was associated with HER2-amplified patients in neoadjuvant treatment. However, the relationship between the HER2 gene amplification level and pCR to concurrent neoadjuvant therapy is still unclear. A study from Yu found a better pCR rate with both a higher HER2/CEP17 ratio and a higher HER2 copy number and then demonstrated that HER2-amplified breast cancer was sensitive to NAC (9). This conclusion was also confirmed in trastuzumab-treated HER2-amplified patients from neoadjuvant trials by Wu (10). A recent study suggested that proteomic changes after a single cycle of HER2-targeted therapy could help identify tumors that would eventually undergo a pCR, validated a single marker CD45 as a classifier that robustly predicted pCR, and indicated that CD45-positive cell counts measured by conventional immunohistochemistry had similar results (11). These findings indicate that screening predictive markers in neoadjuvant treatment and the application of predictive models will have guiding significance for adjusting subsequent therapies. Therefore, there is an urgent need for pCR predictors in medicine. Some studies showed that pCR is predictive for long-term outcomes in HER2-positive and triple-negative breast cancer (12). Markers with predictive value can improve the probability of predicting pCR and also provide a reference for the follow-up treatment after neoadjuvant therapy. At present, studies performed in neoadjuvant treatment with trastuzumab and pertuzumab are still insufficient.

The immune system has become a promising new target for the diagnosis of breast cancer. Tumor-infiltrating lymphocytes (TILs) are included in the microenvironment of breast cancer, and the levels of TILs may also be considered prognostic factors (13). TILs consist of different cells such as CD3+ TILs, CD4+ TILs, CD8+ TILs, and FoxP3+ TILs (14). Treg cells induce the inactivation of CD8+ TILs, which plays a part in causing the death of tumor cells (15). A recent study reported that a high ratio of CD8+TILs to FOXP3+ regulatory T cells significantly improved patient survival in breast cancer (16). In our previous study, the original intention of measuring CD8 levels was to screen the immunomodulatory (IM) type in triple-negative breast cancer (TNBC). However, we found that CD8+ TILs in our patients could be used as a robust marker for predicting the rate of pCR to neoadjuvant treatment, considering the HER2/CEP17 ratio and histological grade. Although some studies have shown that TILs might have significant predictive values for NAC response (17) and indicate good survival in the adjuvant setting (18), no proven results of their roles in predicting the pCR rate in a neoadjuvant treatment setting have been found.

In summary, previous studies revealed that pCR was closely correlated with the HER2/CEP17 ratio in neoadjuvant anti-HER2 dual-targeted treatment (19). A few studies have shown the relationship between CD8 levels and response in dual-targeted neoadjuvant therapy with trastuzumab and pertuzumab. In this study, we investigated the relationship among HER2 amplification, CD8 levels, and pCR to find a better predictive value for HER2-amplified patients.

## Patients and Methods

### Patients

**Figure 1:**
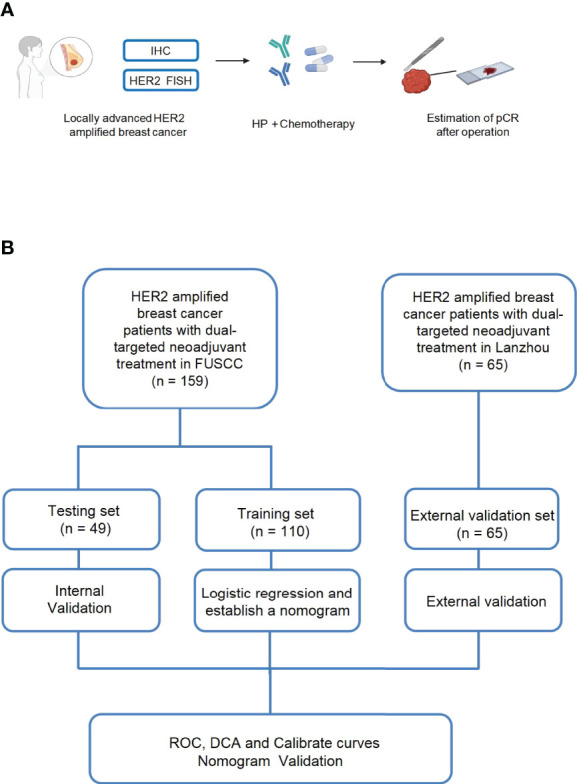
Schematic illustration of our experimental design **(A, B)**. One hundred fifty-nine patients with locally advanced HER2-amplified breast cancer, histologically confirmed by core needle biopsy, were selected. HER2-amplified patients were defined as those who were 2+ or 3+ positive for HER2 overexpression by immunohistochemistry (IHC) or HER2 amplified by fluorescence *in situ* hybridization (FISH) and received neoadjuvant treatment with pertuzumab and trastuzumab plus chemotherapy. Then, pCR was estimated after the surgery.

**Figure 2:**
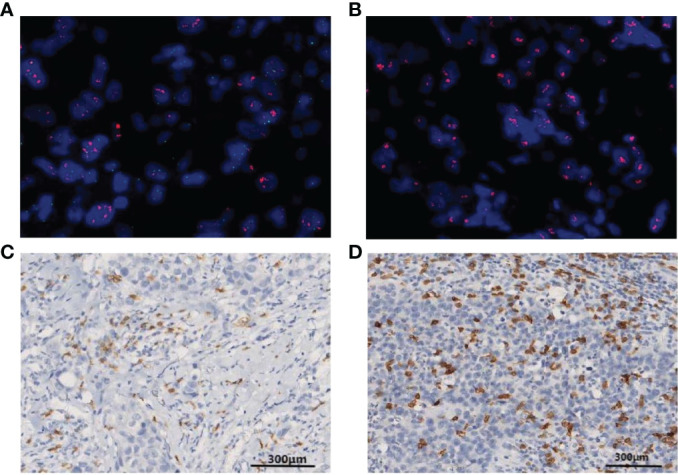
Fluorescence *in situ* hybridization (FISH) is shown for HER2 gene amplification in specimens and in corresponding histological sections (HER2, red signal; CEP17, green signal). **(A)** A patient with positive HER2 amplification status of a HER2/CEP17 ratio <8. **(B)** A patient with positive HER2 amplification status of a HER2/CEP17 ratio ≥8. **(C)** Patterns of CD8+ T-lymphocyte IHC staining >10% CD8 IHC staining (100× magnification). **(D)** More than 20% CD8 IHC staining (100× magnification).

We retrospectively selected 159 consecutive patients with HER2-amplified breast cancer, histologically confirmed by core needle biopsy, who received neoadjuvant treatment with pertuzumab and trastuzumab plus chemotherapy in the Department of Breast Surgery, Fudan University Shanghai Cancer Center (FUSCC, Shanghai, China), from January 1, 2020 to June 30, 2021. Furthermore, we also collected an external validation set from the First Hospital of Lanzhou University (Lanzhou, Gansu, China), including 65 patients treated in the same way and during the same period. HER2-amplified patients were defined as those 2+ or 3+ positive for HER2 over-expression by IHC and amplified by fluorescence *in situ* hybridization (FISH) (**Figures 1**, **2**). The detailed inclusion criteria were as follows: the absence of metastases, as assessed using chest computed tomography (CT) scan, bone scan, and positron emission tomography (PET)/CT; normal hematopoietic, hepatic, and renal functions; and a normal echocardiogram without severe cardiac arrhythmia or heart failure. The exclusion criteria were a previous malignancy history (except for inactive non-melanoma skin cancer and *in situ* carcinoma of the cervix), grade 2 or higher neurotoxicity according to the National Cancer Institute Common Toxicity Criteria (version 3.0) (http://ctep.cancer.gov), active infection, and other comorbid conditions that would affect drug tolerance or impair compliance. The studies involving participants were reviewed and approved by the independent ethics committee/institutional review board of The First Hospital of Lanzhou University Ethical Committee and Fudan University Shanghai Cancer Center Ethical Committee. Written informed consent was provided by all patients.

### Treatment

Invasive carcinomas were diagnosed by core needle biopsy in all patients. Ultrasound, breast magnetic resonance imaging (MRI), and CT scans of the chest and abdomen were conducted for clinical staging. Fine needle aspiration cytology was performed when metastasis to axillary lymph nodes was suspected or found. All patients received carboplatin [area under curve (AUC)=6] and docetaxel [75 mg/m^2^, without dose escalation] plus an 8-mg/kg loading dose of trastuzumab, followed by a 6-mg/kg maintenance dose, and an 840-mg pertuzumab loading dose followed by a 420-mg maintenance dose every 3 weeks for 6 cycles (the TCbHP regimen). No patient required reducing the dosage owing to tolerance difficulties (8).

### Immunohistochemistry

IHC assessment was carried out with paraffin-embedded tumor samples biopsied prior to the neoadjuvant treatment. Estrogen receptor (ER), progesterone receptor (PgR), the Ki67 labeling index, and HER2 expression were evaluated by IHC in all patients (20). For comparison, each HER2-amplified tumor was ultimately evaluated based on the updated 2018 American Society of Clinical Oncology (ASCO)/College of American Pathologists (CAP) Clinical Practice Guidelines as follows (1): IHC 3+ positive, circumferential complete, intense membrane staining in >10% of tumor cells (2); IHC 2+ equivocal, weak to moderate complete membrane staining in >10% of tumor cells, moderate to intense but incomplete (basolateral or lateral) staining, or circumferential intense staining in ≤10% of cells (3); IHC 1+ negative, incomplete membrane staining that is faint/barely perceptible in >10% of tumor cells; and (4) IHC 0 negative, no staining or incomplete, faint/barely perceptible staining in ≤10% of tumor cells (21). Tumors were positive for ER and PgR, with ≥1% of cells being positive (22). IHC for CD8 levels and androgen receptor (AR) staining was performed on the tumors of all patients. The proportion of CD8+ T cells was calculated as the percentage of CD8+ T cells among total cells. Any cell with CD8-positive staining was counted as CD8+ TILs. Results were recorded as percentage of IHC-stained cells and adjudicated by two pathologists (23). The following primary antibodies were used: anti-AR (Abcam, ab133273), anti-CD8 (SP57, Ventana), anti-ER (Abcam, ab108398), and anti-PgR (Abcam, ab16661).

### HER2 FISH

The analysis of FISH was carried out on deparaffinized 5-μm tissue sections using the Path Vysion HER2 DNA probe kit and the HER2/CEP17 probe mixture (Abbott Molecular, Des Plaines, IL, USA) (24). Experienced pathologists in the Department of Pathology of FUSCC and the Department of Pathology of the First Hospital of Lanzhou University assessed the FISH. The pathologists reported the average copy numbers of HER2 and CEP17 and the HER2/CEP17 ratio for each patient. The standard College of American Pathologists and the American College of Medical Genetics guidelines were used to assess the quality control of the HER2 FISH test routinely (25). All samples in our study were HER2 amplified with a HER2/CEP17 ratio ≥ 2.0 (26).

### Evaluation of Clinical Response and Pathological Response

The modified Response Evaluation Criteria in Solid Tumors (RECIST1.1) criteria defined the clinical response (27). The semi-quantitative Miller–Payne grading system was used to measure pathological response. This measures the reduction in percentage in invasive tumor volume and cellularity based on the pathological assessment of surgical samples after neoadjuvant treatment (28, 29). In our study, pCR was defined both in the breast (grade 5 of the Miller–Payne score) and axillary regions.

### Construction and Validation of a Nomogram Model

All the included samples were assigned into a training set and a testing set randomly according to a ratio of 7:3 based on an approach of splitting the data by the hospitalization date (30). We used an independent cohort (n=65) from the First Hospital of Lanzhou University as an external validation set. We selected the HER2/CEP17 ratio, CD8 levels, and histological grade as variables, and used the logistic regression to construct a nomogram model in training set (31). The fit of the model was assessed by the Hosmer–Lemeshow goodness-of-fit chi-square test. Each variable was assigned a point value by drawing a vertical line between the appropriate variable value and the scale in the first row. We can get the total nomogram score by summing up the scores for each of the variables. Then, a probability of pCR can be determined by drawing a vertical line between the total score and the scale in the last row. The area under the curve (AUC) of the receiver operating characteristic (ROC) curve, calibration curves, and decision curve analysis (DCA) were employed to evaluate the nomogram’s performance.

### Statistical Analysis

General characteristics of study subjects were presented as the mean ± standard deviation (SD) for continuous variables and the number (percentage) for categorical variables. Categorical data were compared using the chi-square or Fisher’s exact test. We used the logistic regression to obtain the odds ratio (OR) with 95% confidence interval (CI) for the association with the response. ROC curve analysis was used to assess the prediction power of the HER2/CEP17 ratio, CD8 levels, and histological grade. For all analyses, a *p*-value <0.05 was considered significant. All statistical analyses were carried out in R (version 3.6.3). In addition, “glm,” “rms,” “pROC,” “Calibration Curves,” and “Decision Curve” packages were used.

## Results

### Patient Characteristics

We recruited 159 eligible HER2-amplified patients with locally advanced breast cancer from January 2020 to June 2021, and 142 of them were premenopausal. All of the patients received TCb plus trastuzumab and pertuzumab as neoadjuvant treatment for six cycles. Clinicopathological characteristics are shown in **Table 1**. The female patients had a mean age of 49 years (SD=9.852). The number of pretreatment patients in tumor stage T1–2 was 132 (83.2%), and in tumor stage T3–4, it was 27 (16.8%). The histological grades were GI–II (n=61, 38.4%) and GIII (n=98, 61.6%). The Ki67 value was over 20% in most patients (n=150, 94.3%). A total of 113 patients were positive for lymph node metastasis (pretreatment) (71.1%), 46.5% were ER positive, 34.0% were PgR positive, and 40.3% were over 80% AR positive. In the pretreatment FISH HER2 assessment, the mean HER2/CEP17 ratio was 7.8 (SD=3.187), and the mean HER2 copy number was 18.6 (SD=6.313). The number of CD8 levels was 11.4% (SD=8.964). There were 145 (91.2%) posttreatment patients in tumor stage T1–2 and 14 (8.8%) in tumor stage T3–4. One hundred thirty-two patients were negative for lymph node metastasis after treatment (83.0%) **(** **Table 1****)**.

**Table 1:** Patient characteristics according to pathological complete response.

Characteristic	Non-pCR (n = 52)	pCR (n = 107)	All (n = 159)
	Number (%)	Number (%)	Number (%)
Age (years), mean ± SD	47 (10.619)	51 (9.194)	49 (9.852)
HER2 copy number, mean ± SD	16.3 (6.847)	19.8 (5.715)	18.6 (6.313)
HER2/CEP17 ratio, mean ± SD	6.5 (3.141)	8.4 (3.001)	7.8 (3.187)
CD8 levels (%), mean ± SD	8.5 (5.702)	12.8 (9.872)	11.4 (8.964)
Tumor stage (pretreatment)			
cT1–2	43 (82.7)	89 (83.2)	132 (83.2)
cT3–4	9 (17.3)	18 (16.3)	27 (16.8)
Lymph node status (pretreatment)			
Negative	19 (36.5)	27 (25.2)	46 (28.9)
Positive	33 (63.5)	80 (74.8)	113 (71.1)
Grade			
I–II	31 (59.6)	30 (28.0)	61 (38.4)
III	21 (40.4)	77 (72.0)	98 (61.6)
Ki67			
<20%	3 (5.8)	6 (5.6)	9 (5.6)
≥20%	49 (94.2)	101 (94.4)	150 (94.3)
ER status			
Negative	25 (48.1)	60 (56.1)	85 (53.5)
Positive	27 (51.9)	47 (43.9)	74 (46.5)
PgR status			
Negative	27 (51.9)	78 (72.8)	105 (66.0)
Positive	25 (48.1)	29 (27.2)	54 (34.0)
HER2 status IHC score			
2+	10 (19.2)	10 (9.3)	20 (12.6)
3+	42 (80.8)	97 (90.7)	139 (87.4)
AR			
<80%	12 (23.1)	22 (20.6)	34 (21.4)
≥80%	18 (34.6)	46 (43.0)	64 (40.3)
Unknown	22 (42.3)	39 (36.4)	61 (38.3)
Tumor stage (post-treatment)			
cT1–2	38 (73.1)	107 (100)	145 (91.2)
cT3–4	14 (26.9)	0 (0)	14 (8.8)
Lymph node status (post-treatment)			
Negative	25 (48.1)	107 (100)	132 (83.0)
Positive	27 (51.9)	0 (0)	27 (17.0)
Miller–Payne score			
Grades 1–4	33 (63.5)	0 (0)	33 (20.8)
Grade 5	19 (36.5)	107 (100)	126 (79.2)

Data are presented as the number (percentage) of patients unless otherwise stated. Miller-Payne score: progression, off study prior to surgery for clinical progression or additional non-protocol therapy; grade 1, no or minimal reduction in tumor; grade 2, up to 30% reduction; grade 3, 30%–90% reduction; grade 4, 90% reduction but with some residual invasive (or axillary) disease; grade 5, no residual invasive carcinoma (or axillary) metastasis.

AR, androgen receptor; ER, estrogen receptor; HER2, human epidermal receptor 2; IHC, immunohistochemistry; pCR, pathological complete response in the breast and axilla; PgR, progesterone receptor; SD, standard deviation.

### Treatment Response and Predictors of Response

In univariate logistic regression analyses in the training set, the HER2/CEP17 ratio (OR=1.307, *p*=0.001, 95% CI: 1.117–1.530), HER2 copy number (OR=1.097, *p*=0.012, 95% CI: 1.021–1.180), and CD8 level (OR=1.072, *p*=0.024, 95% CI: 1.009–1.139) (**Table 2**) were better associated with pCR. Regarding other predictors of pCR, PgR positivity and histological grade had a significant effect on pCR in the breast and axilla. Nevertheless, tumor size, Ki67, lymph node status, and ER status at baseline had no significant effect on tumor response. In the univariate analysis, compared with pCR, the HER2/CEP17 ratio had a higher OR value than the HER2 copy number. The HER2 copy number was excluded from the multivariate analysis to avoid confounding.

**Table 2:** Univariate and multivariate logistic regression models predicting pathological complete response after neoadjuvant treatment in training set.

Characteristics	Univariate			Multivariate		
	OR	95% CI	*p*	OR	95% CI	*p*
Age	1.048	1.003–1.095	0.038	1.026	0.972–1.083	0.349
HER2 copy number	1.097	1.021–1.180	0.012	NA		
HER2/CEP17 ratio	1.307	1.117–1.530	0.001	1.409	1.148–1.729	0.001
CD8 levels	1.072	1.009–1.139	0.024	1.117	1.034–1.208	0.005
Tumor stage				NA		
cT1–2	1.000					
cT3–4	0.596	0.193–1.835	0.367			
Lymph node status				NA		
Negative	1.000					
Positive	1.625	0.691–3.823	0.266			
Grade						
I–II	1.000			1.000		
III	3.200	1.374–7.454	0.007	4.068	1.468–11.277	0.007
Ki67				NA		
<20%	1.000					
≥20%	1.177	0.205–6.766	0.855			
ER				NA		
Negative	1.000					
Positive	0.647	0.284–1.473	0.300			
PgR						
Negative	1.000			1.000		
Positive	0.377	0.163–0.872	0.023	0.664	0.221–1.996	0.466
HER2 status IHC score				NA		
2+	1.000					
3+	2.222	0.685–7.213	0.184			
AR				NA		
<80%	1.000					
≥80%	0.801	0.281–2.283	0.678			

AR, androgen receptor; CI, confidence interval; ER, estrogen receptor; HER2, human epidermal receptor 2; IHC, immunohistochemistry; OR, odds ratio; PgR, progesterone receptor. NA, not applicable.

Similarly, in the multivariate analysis, women were more likely to achieve pCR if tumors showed a higher HER2/CEP17 ratio (continuous) (OR=1.409, *p*=0.001, 95% CI: 1.148–1.729). CD8 levels (continuous) were predictive of pCR, with an OR of 1.117 (*p*=0.005, 95% CI: 1.034–1.208). The probability of achieving pCR was also depended on histological grades (OR=4.068, p=0.007, 95% CI: 1.468–11.277). Next, the multivariate analysis revealed that PgR positivity (OR=0.664, *p*=0.466, 95% CI: 0.221–1.996) was not associated with pCR (**Table 2**). We modeled the HER2/CEP17 ratio and CD8 levels as continuous variables and histological grade as a categorical variable.

### Validation of a Predictive Nomogram Model

**Figure 3:**
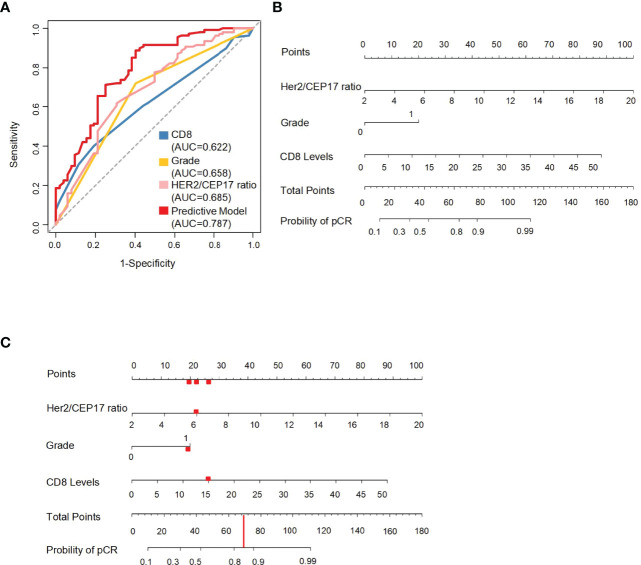
The ROC curves of the HER2/CEP17 ratio, CD8 levels, and histological grade and the predictive nomogram model. **(A)** ROC curves constructed using the HER2/CEP17 ratio, CD8 levels, and histological grade in the whole cohort. **(B)** Nomogram to predict the probability of pathological complete response (pCR). HER2/CEP17 ratio, CD8 levels, and histological grade (0 present grade I–II, 1 present grade III) in the training set. **(C)** The red line and dots demonstrate usage of the model: a patient of HER2-amplified breast cancer with histological grade 3 and 15% of CD8 levels, and HER2/CEP17 ratio is 6 would have a total of 70 points (20 for histological grade 3, 22 for HER2/CEP17 ratio, and 28 for CD8 levels). The predictive value of pCR after NAC for this patient is 85%.

An ROC curve was constructed using the HER2/CEP17 ratio, CD8 levels, and histological grade to select the best responders to concurrent NAC with trastuzumab and pertuzumab. The AUC was 0.787 in the whole cohort. The AUC of the HER2/CEP17 ratio, CD8 levels, and histological grade was 0.685, 0.622, and 0.658, respectively, in the whole cohort (**Figure 3A**). We integrated pCR with the HER2/CEP17 ratio, CD8 levels, and histological grade to construct a prognostic nomogram model in training set (**Figures 3B** **, C**).

**Figure 4:**
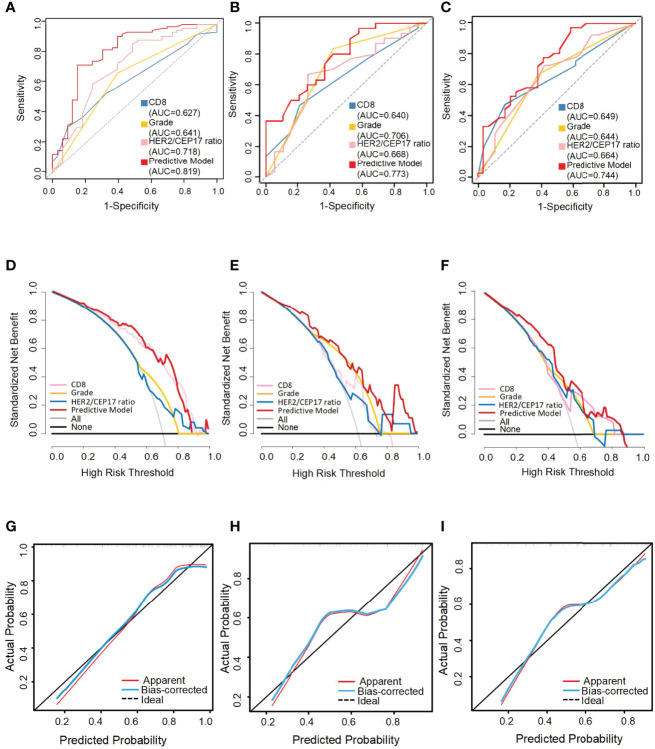
The receiver operating characteristic (ROC) curves, decision curve analysis (DCA), and calibration curve for the predictive nomogram model in the training set, testing set, and external validation set. AUC, area under the curve. The ROC curves of the training set **(A)**, the testing set **(B)**, and the external validation set **(C)**. Decision curve analysis (DCA) of the training set **(D)**, testing set **(E)**, and external validation set **(F)**. Calibration curve of the nomogram in the training set **(G)**, the testing set **(H)**, and the external validation set **(I)**.

With this nomogram model, the AUC value was 0.819 in the training set and 0.773 and 0.744, respectively, in the testing and external validation sets. The AUC of the HER2/CEP17 ratio, CD8 levels, and histological grade was 0.718, 0.627, and 0.641 in the training set (**Figure 4A**). The AUC of the HER2/CEP17 ratio, CD8 levels and histological grade was 0.668, 0.640, and 0.706 in the testing set (**Figure 4B**). The AUC of the HER2/CEP17 ratio, CD8 levels, and histological grade was 0.664, 0.649, and 0.644, respectively, in the external validation set (**Figure 4C**). From the results above, it can be seen that the results of the testing set and external validation set were generally consistent with the training set, and the combined AUC value of the HER2/CEP17 ratio, CD8 levels, and histological grade was better than the individual indicators.

The performance of the nomograms was validated with ROC curve, calibration curve, and DCA for discriminative ability, accurate prediction, and clinical utility, respectively. The y-axis represents the net benefit in DCA curves. The red line indicates the predictive nomogram. The gray line represents the assumption that all patients obtain pCR. The horizontal black line represents the assumption that no patients get pCR. DCA was to show the clinical benefits of the nomogram in the training set, testing set, and external validation set. As was shown in **Figure 4**, DCA curves showed the clinical net benefits in the training set (**Figure 4D**), testing set (**Figure 4E**), and the external validation set (**Figure 4F**). The DCA curve of the predictive model (red) revealed better beneficial than the HER2/CEP17 ratio (pink), CD8 levels (blue), and histological grade (gold), respectively, in different sets. The calibration curve of the nomogram showed good agreement between the actual observations and the predicted outcomes in the training set (**Figure 4G**), testing set (**Figure 4H**), and external validation set (**Figure 4I**). As the prediction curves were close to the standard curve (Y=X), the final model had good performance and high applicability.

## Discussion

In the neoadjuvant setting, dual-targeted treatment by trastuzumab and pertuzumab is standard for HER2 gene-amplified breast cancer. In the TRYPHAENA and NeoSphere study, the improvement of the pCR rate was confirmed by pertuzumab with trastuzumab and standard chemotherapy combined (7, 8). At present, studies evaluating the therapeutic effect are still insufficient. The existing indicators are used for single-targeted therapy but not dual-targeted therapy. Our study revealed that the HER2/CEP17 ratio, CD8 levels, and histological grade were associated with the response to the same neoadjuvant treatment. There are some potential reasons why HER2-amplified patients are more sensitive to dual-targeted therapy. First, trastuzumab induces internalization and degradation of the HER2 receptor by binding to the extracellular domain of the transmembrane HER2 receptor (32). Second, trastuzumab is related to immune and antibody-dependent cell-mediated cytotoxicity (ADCC). FCγR polymorphisms play a part in trastuzumab-mediated ADCC and also predict the clinical results in breast cancer patients (33). When trastuzumab binds to the FCγR of natural killer cells, it triggers ADCC and activates cell lysis (10). Lastly, trastuzumab leads to the inhibition of its downstream signaling *via* the RAS/MAPK and PI3K/AKT pathways, ultimately suppressing cellular growth and proliferation signaling. Pertuzumab prevents potent ligand-dependent HER2/HER3 heterodimerization, suppressing downstream PI3K and MAPK pathways (34, 35). The combination of the two drugs in chemotherapy should be taken into consideration for their contribution to the improved pCR outcome of NAC. Some studies in HER2-amplified breast cancer patients receiving neoadjuvant treatment focused on the level of HER2 amplification and its relationship with pCR. In a study conducted in South Korea, the HER2/CEP17 ratio was related with pCR in dual-targeted HER2 neoadjuvant treatment (19). Another study demonstrated that a higher HER2/CEP17 ratio was predictive of pCR to NAC in patients included in the National Cancer Database (36). Regarding the predictive value, we found that, compared with the HER2 copy number, the HER2/CEP17 ratio was more closely associated with pCR outcome, which agrees with previous findings. Kogawa showed a positive correlation between the HER2/CEP17 ratio and pCR (37). However, it is not reliable enough to depend on a single indicator as a predictor, so we built a combined model of the HER2/CEP17 ratio, CD8 level, and histological grade, which has a better predictive value for pCR.

Tumor histological grade refers to the degree of tumor tissue anaplasticity, including tumor differentiation, arrangement, number of mitoses, and local infiltration of cancer cells. The degree of tumor tissue anaplasticity can provide a reference basis for clinical treatment and prognosis estimation. The histological grade was associated with pCR in our research. Our findings are consistent with existing findings. A study from Rouzier found that the histological grade after the completion of neoadjuvant treatment was a good independent prognostic factor for pCR in patients with breast cancer (38). The correlation between pCR and long-term outcome was higher in patients with high-grade tumors than in those with low-grade tumors in neoadjuvant treatment (12). Furthermore, another article concludes the relationship between histological grade and neoadjuvant dual-target and chemotherapy response pCR in HER2-amplified tumors. Their analysis of the HER2-amplified tumors confirmed that a higher pCR rate was seen in histological grade III tumors treated with NAC and dual anti-HER2 therapy (39), which was consistent with our conclusion.

The immune microenvironment may affect dual-targeted neoadjuvant treatment efficacy. TILs can produce a positive effect on the frequency of pCR in TNBC and HER2-amplified breast cancer (13, 40). One study showed that high pre-NAC TIL levels were clearly predictive of pCR and can act as a surrogate marker to predict therapeutic effects of a dual-targeted neoadjuvant treatment regimen for HER2-amplified breast cancer (41). For multivariate analysis, our study found that CD8 levels could act as a robust marker to predict the pCR rate to neoadjuvant treatment, considering the HER2/CEP17 ratio and histological grade. Research has shown that an immunological signature consisting of the presence of a high number of CD8+TILs on final surgical biopsy samples of breast tumors treated with NAC is associated with pCR (42). Another possible reason is that the cell death induced by chemotherapy can lead to the release of tumor antigens, which can be processed by antigen-presenting cells (APCs) to CD8+ TILs, resulting in the death of cancer cells by activating CD8+ TILs (43).

Some data confirmed the hypothesis that breast cancer is immunogenic and may be targetable by immune-modulating therapies. CD8+ TILs play essential roles in the immune microenvironment, mainly by killing tumor cells through cytotoxic effects. TILs as an indicator of pre-existing immunogenicity might be useful for further stratification of breast cancer in clinical trials, including chemotherapy, anti-HER2 therapies, and future combinations with immune therapies (13). Trastuzumab also attracts cytotoxic innate immune cells to the tumor microenvironment, a concept commonly known as ADCC.

At the same time, we found that the HER2/CEP17 ratio, histological grade, and CD8 levels were positively associated with pCR, so we used an ROC curve to construct a predictive model for pCR with these three markers to select the best responder to concurrent NAC with trastuzumab and pertuzumab. It is noteworthy that in this model of neoadjuvant treatment, we confirm that the combined AUC value of the HER2/CEP17 ratio, CD8 levels, and histological grade was better than the individual indicators, which showed that an immunological effect partially mediates the predictive impact of neoadjuvant treatment. This study will provide more options for the clinical treatment of HER2-amplified breast cancer. The pCR prediction models were composed of different factors, which were currently widely studied, and there was no gold standard for models. The advantage of our prediction model was that we used HER2/CEP17 ratio and histological grade as two factors because they were recognized factors that were associated with pCR in HER2-positive breast cancer, and we also added CD8+TILs immune factor as one of the indicators. The AUC of the HER2/CEP17 ratio, CD8 levels, and histological grade was 0.718, 0.627 and 0.641, respectively, and the AUC was 0.819 of the combined model in the training set. Of course, this conclusion needs to be further verified by larger samples in the future.

We analyzed why hormone receptors (HRs) status is not predictive of pCR in our study and found that the predictive value of HR status remains unclear in HER2-amplified breast cancer. Previous studies demonstrated that HER2-amplified/hormone receptors-negative patients treated with the TCbHP regimen had higher pCR rates (71.1%–76.2%) (44, 45). However, in our study, ER and PgR status at baseline had no significant effect on tumor response (**Table 2**). Other studies also showed that the ER and PgR status was probably not a predictor for pCR (46), and the ER status was not predictive of pCR in subgroup analyses in HER2-amplified breast cancer patients (11). The small sample size perhaps is another explanation to our findings. Some potential factors were not found to be significant in our current study due to this reason. Prospective and randomized cohort study with larger sample size was needed to validate the predictive value of hormone status, and multi-omics profiling might facilitate the understanding of the effects of hormone status on dual-targeted therapy.

There are also some limitations in our study. First, studies conducted with larger sample sizes are required, and more clinical studies are needed to confirm our conclusions further. Second, a single IHC marker often provides inaccurate and incomplete prognostic indices, and more IHC markers should be included to predict pCR in dual-targeted neoadjuvant treatment. This study selected CD8 levels as the predictive marker because of its previous studies, which showed that CD8+TILs were associated with a positive effect of pCR in HER2-amplified breast cancer (47). Our advantage is that these model building indicators can be easily evaluated by pathologists, compared with multiplex techniques or proteomics. Thus, it is easy to apply this model in the clinical setting.

Consequently, it is crucial to find more effective molecular biological indicators to formulate individualized treatment plans and provide precision treatment for HER2-amplified breast cancer patients.

## Conclusion

In conclusion, the HER2/CEP17 ratio, histological grade, and CD8 levels were positively associated with pCR. Furthermore, we constructed a nomogram model that could significantly predict the pCR rate of dual-targeted neoadjuvant treatment in HER2-amplified breast cancer patients.

## Data Availability Statement

The original contributions presented in the study are included in the article/**Supplementary Material**. Further inquiries can be directed to the corresponding authors.

## Ethics Statement

The studies involving participants were reviewed and approved by the independent ethics committee/institutional review board of The First Hospital of Lanzhou University Ethical Committee and Fudan University Shanghai Cancer Center Ethical Committee. The patients/participants provided their written informed consent to participate in this study. Written informed consent was obtained from the individual(s) for the publication of any potentially identifiable images or data included in this article.

## Author Contributions

Conception/design: YX, KY, and XL. Provision of study material or patients: YX. Collection and/or assembly of data: YX and DM. Data analysis and interpretation: JD and SC. Manuscript writing: YX and JD. Final approval of manuscript: YX, JD, DM, SC, KY, and XL. All authors contributed to the article and approved the submitted version.

## Funding

This study was funded by the Science and Technology Planning Project of Gansu Province of China (21JR7RA371), the fund of The First Hospital of Lanzhou University of Gansu Province of China (ldyyyn2020-91), and The Wu Jieping Medical funding of China (320.6750.2021-10-100). The fund of Lanzhou Science and Technology Bureau of China (2022-ZD-96). The funders had no role in the design of the study and collection, analysis, interpretation of data, or writing of the manuscript.

## Conflict of Interest

The authors declare that the research was conducted in the absence of any commercial or financial relationships that could be construed as a potential conflict of interest.

## Publisher’s Note

All claims expressed in this article are solely those of the authors and do not necessarily represent those of their affiliated organizations, or those of the publisher, the editors and the reviewers. Any product that may be evaluated in this article, or claim that may be made by its manufacturer, is not guaranteed or endorsed by the publisher.
